# Nucleus Accumbens Associated Protein 1 in Cancers—The Real Value

**DOI:** 10.3390/ijms252413632

**Published:** 2024-12-20

**Authors:** Marlena Janiczek-Polewska, Tomasz Kolenda, Paulina Poter, Inga Jagiełło, Joanna Kozłowska-Masłoń, Katarzyna Regulska, Julian Malicki, Andrzej Marszałek

**Affiliations:** 1 Department of Clinical Oncology, Greater Poland Cancer Center, 61-866 Poznan, Poland; 2 Department of Electroradiology, Poznan University of Medical Sciences, 61-701 Poznan, Poland; 3 Research and Implementation Unit, Greater Poland Cancer Centre, 61-866 Poznan, Poland; 4 Department of Clinical Pathology, Poznan University of Medical Sciences, Greater Poland Cancer Center, 61-866 Poznan, Poland; 5 Laboratory of Cancer Genetics, Greater Poland Cancer Centre, 61-866 Poznan, Poland; 6 Institute of Human Biology and Evolution, Faculty of Biology, Adam Mickiewicz University, 61-712 Poznan, Poland; 7 Pharmacy, Greater Poland Cancer Centre, 61-866 Poznan, Poland; 8 Department of Clinical Pharmacy and Biopharmacy, Poznan University of Medical Sciences, 61-701 Poznan, Poland

**Keywords:** NACC1, cancers, TCGA, diagnostic, treatment, genetics

## Abstract

Malignant tumors are a leading cause of death worldwide, second only to cardiovascular disease. They occur in every population and have a high risk of mortality. The etiopathogenesis of malignant tumors is diverse and there are still many unknowns, leading to huge diagnostic and therapeutic challenges. Therefore, the search for ideal diagnostic and therapeutic agents is ongoing. One of the promising factors affecting cancer is the nucleus accumbens associated protein 1 (NACC1). It is a transcriptional coregulator. Moreover, it plays a multifaceted role in promoting tumorigenesis. NACC1 expression analyses were performed using The Cancer Genome Atlas (TCGA) data accessed from the University of Alabama at Birmingham Cancer (UALCAN) database, and the expression data were interconnected with clinicopathological parameters. All statistical analyses were conducted using GraphPad Prism and Statistica. The results revealed that NACC1 was expressed in almost all of the analyzed cancers, and its expression level correlates with different clinicopathological parameters. This study demonstrates that NACC1 is potentially involved in the pathogenesis, invasion, and immune response associated with many cancers. However, NACC1 is not a suitable candidate as a diagnostic biomarker as it is not specific for any type of malignancy and there are discrepancies in its expression in relation to many clinicopathological parameters. The implementation of NACC1 as a therapeutic target may improve the effectiveness of cancer treatments.

## 1. Introduction

**Figure 1:**
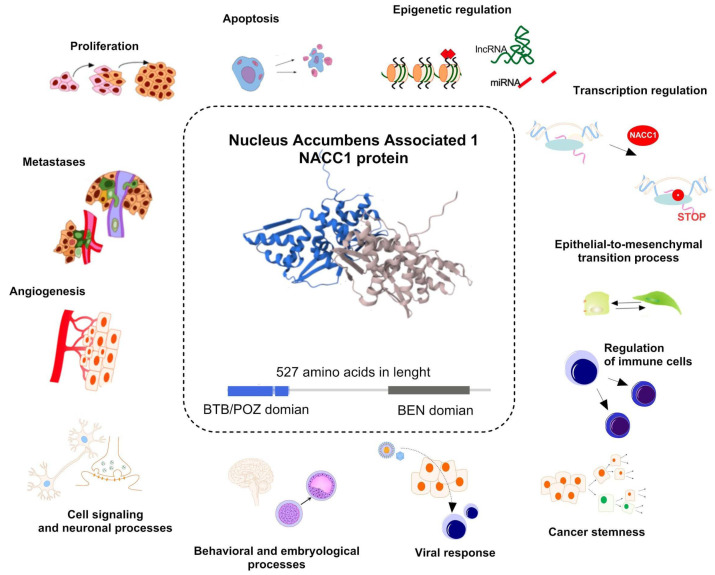
Function of nucleus accumbens associated protein 1 (NACC1) in biological processes. The protein and the NACC1 gene transcript itself play an important role in non-neoplastic processes (cell signaling and neuronal processes, regulation of behavioral and embryological processes, as well as with cellular response to viral infection), but mainly it is closely related to the neoplastic process. In the case of cancers, NACC1 is associated with the regulation of cellular processes (proliferation, angiogenesis, metastasis, apoptosis, as well as regulation of immune cells), epigenetic regulation (regulation of histone, and influence on lncRNA and miRNA), transcription regulation, and regulation of cell phenotype through processes such as epithelial–mesenchymal transition and regulation of cancer stemness. Structure of NACC1 protein was taken from https://www.ebi.ac.uk/pdbe/pdbe-kb/proteins/Q96RE7 (accessed on 10 December 2024) [7,15,27,28,29,30].

Cancer is one of the greatest health issues of the 21st century, being a significant problem in all areas of life [1]. It is primarily a public health problem, but it is also a social and economic one [2,3,4]. Cancer is often diagnosed in very advanced stages, which unfortunately translates into an unfavorable prognosis for patients. Despite very advanced research conducted around the world on the pathogenesis of cancer, we still do not have the appropriate tools for the early diagnosis of patients [5] Cancer causes 16.8% of deaths worldwide. Moreover, it causes one-third of premature deaths among people aged 30–69 [6]. Nucleus accumbens associated protein 1 (NAC1) is a cancer-related transcriptional factor and is encoded by the nucleus accumbens associated protein 1 (NACC1) gene. It is a transcriptional coregulator and plays a multifaceted role in promoting tumorigenesis. Its synonyms include BEND8, BTBD14B, BTBD30, NAC-1, and NAC1. NACC1 is a member of the broad family of tramtrack, bric-a-brac/poxvirus, and zinc finger (BTB/POZ) complex proteins, mainly exerting its biological functions as a transcriptional coregulator [7,8]. BTB/POZ proteins are involved in proliferation, apoptosis, and transcriptional regulation [9]. The encoded protein is a transcriptional repressor and suppresses the transcription of the potential tumor suppressor GADD45G interacting protein 1 (Gadd45GIP1), as well as playing a role in stem cell self-renewal and maintenance of pluripotency. The pseudogene of this gene is located on the short arm of chromosome 9 [7,10,11,12]. In addition, abundant data indicate the overexpression of NACC1 in various cancers, which is associated with cancer progression and high relapse rates, thus potentially indicating poor prognosis [13,14,15,16,17,18,19,20,21,22,23]. NACC1 has been described in the literature as a potential prognostic and diagnostic biomarker for many cancers [24]. Most of the available studies in the literature on NACC1 focus on its expression in a single analyzed tumor, which may suggest its spectacular and specific nature [13,14,15,16,17,18,19,20,21,22,23]. However, in correlation with other tumors, it loses its significance. Function of NACC1 in cancer and biological processes was presented in Figure 1. In our research, we aim to analyze the existing data on the occurrence of NACC1 in various cancers based on The Cancer Genome Atlas (TCGA) databases and correlate them with the available data from publications [25,26]. To our knowledge, this is the first comprehensive analysis of NACC1 from TCGA databases for malignant tumors.

## 2. Results

### 2.1. Expression Levels of NACC1 in Cancers Based on the Cancer Genome Atlas

**Figure 2:**
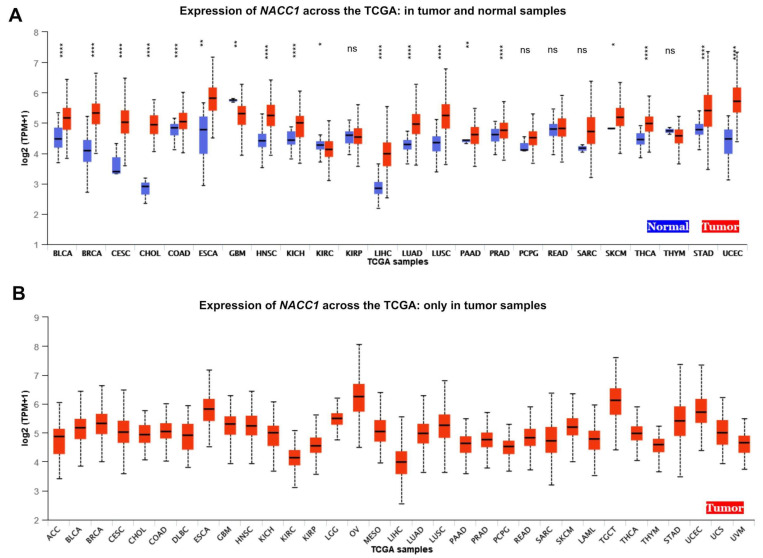
Expression of NACC1 across TCGA cancers (with tumor (red) and normal (blue) samples). (**A**) Expression of NACC1 across the TCGA: in tumor and normal samples. (**B**) Expression of NACC1 across the TCGA: only in tumor samples. Figure taken from the UALCAN database. BLCA, bladder urothelial carcinoma; BRCA, breast invasive carcinoma; CESC, cervical squamous cell carcinoma; CHOL, cholangiocarcinoma; COAD, colon adenocarcinoma; ESCA, esophageal carcinoma; GBM, glioblastoma multiforme; HNSC, head and neck squamous cell carcinoma; KICH, kidney chromophobe; KIRC, kidney renal clear cell carcinoma; KIRP, kidney renal papillary cell carcinoma; LIHC, liver hepatocellular carcinoma; LUAD, lung adenocarcinoma; LUSC, lung squamous cell carcinoma; PAAD, pancreatic adenocarcinoma; PRAD, prostate adenocarcinoma; PCPG, pheochromocytoma and paraganglioma; READ, rectum adenocarcinoma; SARC, sarcoma; SKCM, skin cutaneous melanoma; TGCT, testicular germ cell tumor; THYM, thymoma; THCA, thyroid carcinoma; STAD, stomach adenocarcinoma; UCEC, uterine corpus endometrial carcinoma; ACC, adrenocortical carcinoma; LGG, brain lower-grade glioma; OV, ovarian serous cystadenocarcinoma; MESO, mesothelioma; LAML, acute myeloid leukemia; TGCT, testicular germ cell tumor; UCS, uterine carcinosarcoma; UVM, uveal melanoma; ns, no significant; * *p* < 0.05, ** *p* < 0.01, **** *p* < 0.0001.

Using data from The Cancer Genome Atlas (TCGA), the expression level of NACC1 was determined in 29 cancers, including bladder urothelial carcinoma, breast invasive carcinoma, cervical squamous cell carcinoma, cholangiocarcinoma, colon adenocarcinoma, esophageal carcinoma, glioblastoma multiforme, head and neck squamous cell carcinoma, kidney chromophobe, kidney renal clear cell, liver hepatocellular carcinoma, lung adenocarcinoma, lung squamous cell carcinoma, pancreatic adenocarcinoma, prostate adenocarcinoma, stomach adenocarcinoma, thyroid carcinoma, uterine corpus endometrial carcinoma, kidney renal papillary cell carcinoma, pheochromocytoma and paraganglioma, rectum adenocarcinoma, sarcoma, skin cutaneous melanoma, thymoma, adrenocortical carcinoma, brain lower-grade glioma, mesothelioma, ovarian serous cystadenocarcinoma, and testicular germ cell tumors. The data indicated that NACC1 is expressed in 18 cancers. We considered a *p*-value of <0.05 significant. According to the UALCAN database, we observed a statistically significant expression of NACC1 in the normal vs. primary comparison for bladder urothelial carcinoma, breast invasive carcinoma, cervical squamous cell carcinoma, cholangiocarcinoma, colon adenocarcinoma, esophageal carcinoma, glioblastoma multiforme, head and neck squamous cell carcinoma, kidney chromophobe, kidney renal clear cell, liver hepatocellular carcinoma, lung adenocarcinoma, lung squamous cell carcinoma, pancreatic adenocarcinoma, prostate adenocarcinoma, stomach adenocarcinoma, thyroid carcinoma, and uterine corpus endometrial carcinoma. No differences (*p* > 0.05) were observed for kidney renal papillary cell carcinoma, pheochromocytoma and paraganglioma, rectum adenocarcinoma, sarcoma, skin cutaneous melanoma, and thymoma. No data on adrenocortical carcinoma, brain lower-grade glioma, mesothelioma, ovarian serous cystadenocarcinoma, and testicular germ cell tumors were available. All data are presented in Figure 2 and Table 1.

### 2.2. Tumor Grade

**Figure 3:**
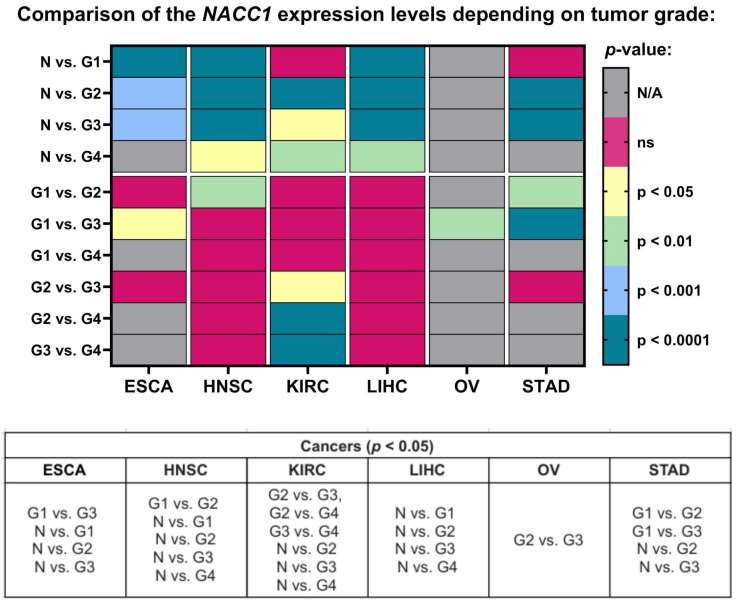
Differences in the expression levels of NACC1 depending on tumor grade in ESCA, HNSC, KIRC, LIHC, OV, and STAD patients based on the TCGA project. Data taken from the UALCAN database. *p* < 0.05 was considered significant. N, normal; N/A, not analyzed; N/A, not analyzed; ns, no significant; G1, grade 1; G2, grade 2; G3, grade 3; G4, grade 4; ESCA, esophageal carcinoma; HNSC, head and neck squamous cell carcinoma; KIRC, kidney renal clear cell carcinoma; LIHC, liver hepatocellular carcinoma; OV, ovarian serous cystadenocarcinoma; STAD, stomach adenocarcinoma.

When we looked into the tumor grade, the expression levels of NACC1 differed in esophageal carcinoma for G1 vs. G3, N vs. G1, N vs. G2, and N vs. G3. In head and neck squamous cell carcinoma, it differed in G1 vs. G2, N vs. G1, N vs. G2, N vs. G3, and N vs. G4. In kidney renal clear cell carcinoma, it differed in G2 vs. G3, G2 vs. G4, G3 vs. G4, N vs. G2, N vs. G3, and N vs. G4. In liver hepatocellular carcinoma, it differed in N vs. G1, N vs. G2, N vs. G3, and N vs. G4. In ovarian serous cystadenocarcinoma, it differed in G2 vs. G3. Finally, in stomach adenocarcinoma, it differed in G1 vs. G2, G1 vs. G3, N vs. G2, and N vs. G3. All data are presented in Figure 3.

### 2.3. Cancer Stage

**Figure 4:**
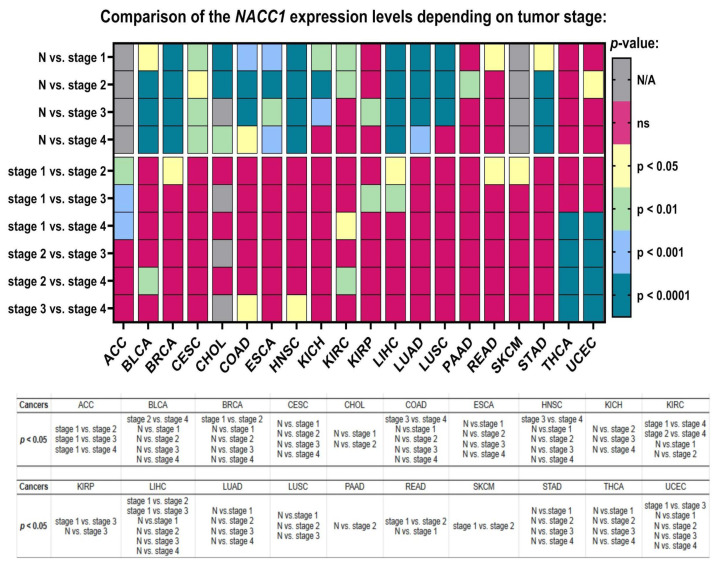
Differences in the expression levels of NACC1 depending on the tumor grade in ACC, BLCQ, BRCA, CESC, CHOL, COAD, ESCA, HNSC, KIRC, and KIRP patients based on the TCGA data. Data taken from the UALCAN database. A *p*-value of <0.05 was considered significant. N, normal; N/A, not analyzed; ns, no significant; ACC, adrenocortical carcinoma; BLCA, bladder urothelial carcinoma; BRCA, breast invasive carcinoma; CESC, cervical squamous cell carcinoma; CHOL, cholangiocarcinoma; COAD, colon adenocarcinoma; ESCA, esophageal carcinoma; HNSC, head and neck squamous cell carcinoma; KICH, kidney chromophobe; KIRC, kidney renal clear cell carcinoma; KIRP, kidney renal papillary cell carcinoma; LIHC, liver hepatocellular carcinoma; LUAD, lung adenocarcinoma; LUSC, lung squamous cell carcinoma; PAAD, pancreatic adenocarcinoma; READ, rectum adenocarcinoma; SKCM, skin cutaneous melanoma; STAD, stomach adenocarcinoma; THCA, thyroid carcinoma; UCEC, uterine corpus endometrial carcinoma.

The expression levels of NACC1 were also associated with the cancer stage in almost all of the analyzed tumors. MESO, OV, PCPG, PRAD, SARC, TGCT, THYM, and SKCM were not analyzed. Moreover, in ACC, a statistical difference in NACC1 expression was demonstrated between stage 1 and the other stages. The expression levels of NACC1 in BRCA, READ, and SKCM differed for stage 1 vs. stage 2. In BLCA, a difference between stage 2 and stage 3 was observed. In COAD and HNSC, it differed in stage 3 vs. stage 4. Moreover, in KIRC, for the stage 1 vs. stage 4 and stage 2 vs. stage 4 comparisons, differences were observed. In KIRP and UCEC, it differed in the stage 1 vs. stage 3 comparison. In LIHC, these differences were in the stage 1 vs. stage 2 and stage 1 vs. stage 3 comparisons. All data are presented in Figure 4.

### 2.4. Race

**Figure 5:**
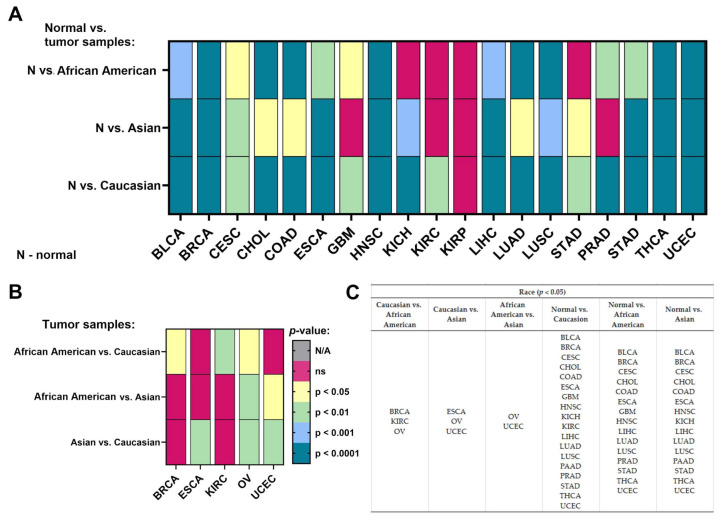
Differences in the expression levels of NACC1 depending on race in the cancers based on the TCGA data: (**A**) in the group normal vs. tumor samples; (**B**) only in the group of tumor samples; (**C**) listed types of tumors in the specified group of comparison. Data taken from the UALCAN database. *p* < 0.05 was considered significant; N, normal; N/A, not analyzed; ns, no significant; BLCA, bladder urothelial carcinoma; BRCA, breast invasive carcinoma; CESC, cervical squamous cell carcinoma; CHOL, cholangiocarcinoma; COAD, colon adenocarcinoma; ESCA, esophageal carcinoma; GBM, glioblastoma multiforme; HNSC, head and neck squamous cell carcinoma; KICH, kidney chromophobe; KIRC, kidney renal clear cell carcinoma; LIHC, liver hepatocellular carcinoma; LUAD, lung adenocarcinoma; LUSC, lung squamous cell carcinoma; OV, ovarian serous cystadenocarcinoma; PAAD, pancreatic adenocarcinoma; PRAD, prostate adenocarcinoma; STAD, stomach adenocarcinoma; THCA, thyroid carcinoma; UCEC, uterine corpus endometrial carcinoma.

We checked the differences in the expression levels of NACC1 depending on the race of the patient for the analyzed cancers. There were three main races, which may play an important role in genetic diversity: Caucasian, African American, and Asian. First of all, we checked the expression levels of NACC1 in normal samples in comparison to tumor samples, divided into the three races. We observed the expression levels of the same tumors in each race, including BLCA, BRCA, CESC, CHOL, COAD, ESCA, HNSC, LIHC, LUAD, LUSC, STAD, THCA, and UCEC. No differences were observed for SARC and KIRP. SKCM was not analyzed, as it was not possible to calculate statistics from the obtained data. MESO, OV, ACC, LGG, and THYM were also not analyzed in this regard. In the Caucasian vs. African American race comparison, we observed differences in the expression of NACC1 in tumors such as BRCA, KIRC, and OV. In the Caucasian vs. Asian race comparison, the expression level of NACC1 was different in ESCA, OV, and UCEC. All data are presented in Figure 5.

### 2.5. Gender

**Figure 6:**
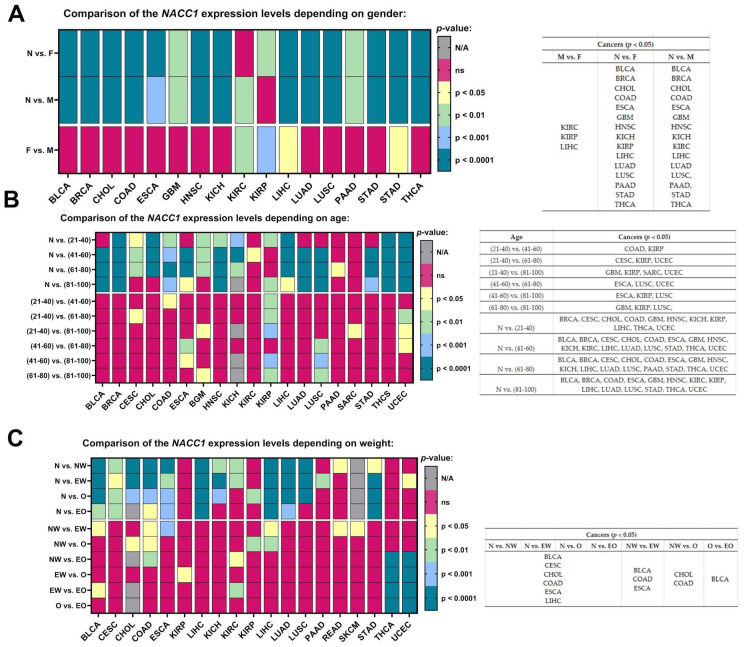
Differences in the expression levels of NACC1 depending on gender (**A**), age (**B**), and weight (**C**) in the cancers based on the TCGA data. Data taken from the UALCAN database. *p* < 0.05 was considered significant; N, normal; N, N/A, not analyzed; ns, no significant; F, female; M, male; EW, extreme weight: BMI greater than or equal to 25 and less than 30; O, obese: BMI greater than or equal to 30 and less than 40; EO, extreme weight: BMI greater than 40; NW, normal weight: BMI greater than or equal to 18.5 and less than 25; BLCA, bladder urothelial carcinoma; BRCA, breast invasive carcinoma; CESC, cervical squamous cell carcinoma; CHOL, cholangiocarcinoma; COAD, colon adenocarcinoma; ESCA, esophageal carcinoma; GBM, glioblastoma multiforme; HNSC, head and neck squamous cell carcinoma; KICH, kidney chromophobe; KIRC, kidney renal clear cell carcinoma; KIRP, kidney renal papillary cell carcinoma; LIHC, liver hepatocellular carcinoma; LUAD, lung adenocarcinoma; LUSC, lung squamous cell carcinoma; PAAD, pancreatic adenocarcinoma; SARC, sarcoma; STAD, stomach adenocarcinoma; THCA, thyroid carcinoma; UCEC, uterine corpus endometrial carcinoma.

We observed the expression levels of NACC1 vs. normal samples with regard to gender in BLCA, BRCA, CHOL, COAD, ESCA, GBM, HNSC, KICH, LIHC, LUAD, LUSC, PAAD, STAD, and THCA. Additionally, we observed that the expression level of NACC1 was higher in males for KIRC, and in females for KIRP, in comparison with normal samples. Furthermore, we observed differences between males and females in KIRC, KIRP, and LIHC tumors. All data are presented in Figure 6A.

### 2.6. Age

Most of the analyzed tumors, regardless of age, showed statistically significant expression levels of NACC1 when compared to normal samples. No differences were observed for PCPG, READ, SARC, and THYM. Moreover, no differences were observed in the oldest age group (i.e., between 81 and 100 years) for CESC, CHOL, KIRC, and PAAD. The expression levels of NACC1 were not assessed for ACC, LGG, MESO, OV, and TGCT. All data are presented in Figure 6B.

### 2.7. Weight

We determined differences for normal samples vs. the NW, EW, O, and EO groups for BLCA, CESC, CHOL, COAD, ESCA, and LIHC tumors. Body weight did not significantly affect the levels in these tumors. Then, the individual tumor groups were analyzed among themselves. The expression levels of NACC1 differed in EW vs. NW in BLCA, COAD, and ESCA. In NW vs. O, differences were observed in COAD and CHOL. Differences were observed between NW and O in KIRP and for EW vs. EO in BLCA. No differences were found in tumors in the O vs. EO group comparison. All data are presented in Figure 6C.

### 2.8. Nodal Metastasis

**Figure 7:**
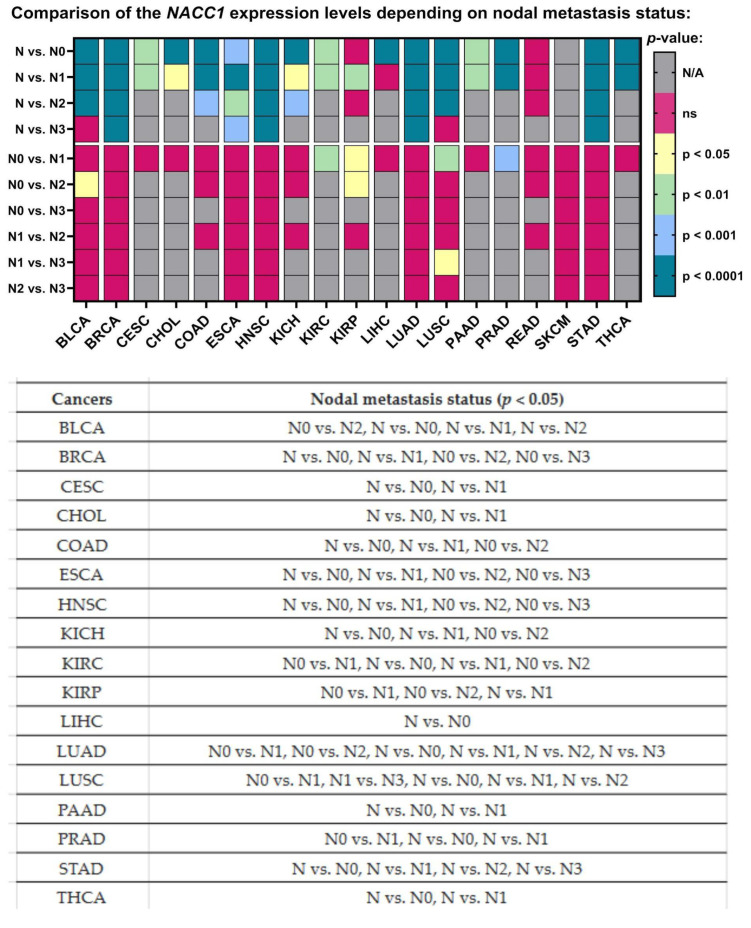
Differences in the expression levels of NACC1 depending on nodal metastasis status in cancers based on the TCGA data. Data taken from the UALCAN database; *p* < 0.05 was considered significant; N, normal; N/A, not analyzed; ns, no significant; N0, no regional lymph node metastasis; N1, metastases in 1 to 3 axillary lymph nodes; N2, metastases in 4 to 9 axillary lymph nodes; N3, metastases in 10 or more axillary lymph nodes; BLCA, bladder urothelial carcinoma; BRCA, breast invasive carcinoma; CESC, cervical squamous cell carcinoma; CHOL, cholangiocarcinoma; COAD, colon adenocarcinoma; ESCA, esophageal carcinoma; HNSC, head and neck squamous cell carcinoma; KICH, kidney chromophobe; KIRC, kidney renal clear cell carcinoma; KIRP, kidney renal papillary cell carcinoma; LIHC, liver hepatocellular carcinoma; LUAD, lung adenocarcinoma; LUSC, lung squamous cell carcinoma; PAAD, pancreatic adenocarcinoma; PRAD, prostate adenocarcinoma; STAD, stomach adenocarcinoma; THCA, thyroid carcinoma.

NACC1 presented different expression levels depending on the number of lymph nodes in almost all the analyzed cancers, in comparison to the normal samples. Moreover, only a few cancers showed a differing expression of NACC1 between nodal metastasis statuses. For NACC1, differences in the expression levels were noted in the following comparisons: N0 vs. N1 for KIRC, KIRP, LUSC, and PRAD; N0 vs. N2 for BLCA and KIRP; and N1 vs. N3 for LUSC. All data are presented in Figure 7.

### 2.9. TP-53 Mutation

**Figure 8:**
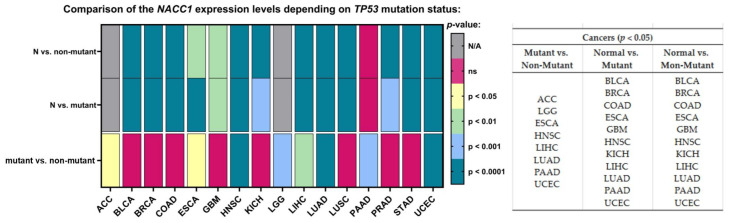
Differences in the expression levels of NACC1 depending on TP-53 mutation status in cancers based on the TCGA data. Data taken from the UALCAN database. A *p*-value of <0.05 was considered significant; N, normal; N/A, not analyzed; ns, no significant;. ACC, adrenocortical carcinoma; BLCA, bladder urothelial carcinoma; LGG, brain lower-grade glioma; BRCA, breast invasive carcinoma; COAD, colon adenocarcinoma; ESCA, esophageal carcinoma; GBM, glioblastoma multiforme; HNSC, head and neck squamous cell carcinoma; KICH, kidney chromophobe; LICH, liver hepatocellular carcinoma; LUAD, lung adenocarcinoma; PAAD, pancreatic adenocarcinoma; UCEC, uterine corpus endometrial carcinoma.

The last parameter—namely the TP-53 mutation status—was associated with differences in the expression level of NACC1 for ACC, LGG, ESCA, HNSC, LIHC, LUAD, PAAD, and UCEC. Moreover, we observed differences for TP-53 mutant vs. normal samples and TP-53 non-mutant vs. normal samples for the same tumors (i.e., BLCA, BRCA, COAD, ESCA, GBM, HNSC, KICH, LIHC, LUAD, PAAD, and UCEC). Sufficient sensitivity and specificity for the TP-53 parameter were not observed in any of the analyzed tumors; therefore, NACC1 expression in tumors is probably independent of TP-53 mutations. All data are presented in Figure 8.

Indeed, statistically, the expression of NACC1 in BLCA, BRCA, COAD, ESCA, GBM, HNSC, Kichc, LiHC, LUD, PAAD, and UCEC was not dependent on TP-53. In comparison, the mutant vs. non-mutant comparison revealed differences in the expression of NACC1 in ACC, LGG, ESCA, HNSC, LIHC, LUD, PAAD, and UCEC. Patients with TP-53 mutation in ACC, LGG, and UCEC tumors presented a difference in NACC1 expression compared with non-mutants. However, only UCEC showed a difference in expression with respect to normal tissue; however, it showed statistical significance when both compared to mutant and non-mutant groups (i.e., it has no sensitivity or specificity for mutant T53).

## 3. Discussion

Cancer is the leading cause of death worldwide. In 2020, it caused almost 10 million deaths; this means that one in six deaths were caused by cancer [14]. These data are alarming. Therefore, new therapeutic and diagnostic factors are being intensively sought to reduce cancer-related mortality [31,32,33,34,35]. There is a significant amount of data in the literature on the presence of NACC1 in various cancers. These data often indicate promising results that could serve as the basis for introducing new diagnostic or therapeutic tools. However, is this really the case? Can one factor be universal for all cancers or, on the contrary, can it perform different functions in individual cancers? In our studies based on the TCGA database, we decided to analyze the function of NACC1 in analyzed cancers. Our results allow us to determine the role of NACC1 in cancers and its possible diagnostic and therapeutic potential.

Analysis of the TCGA database regarding NACC1 expression in tumors revealed that NACC1 overexpression occurred in almost all analyzed tumors. A statistically significant difference in NACC1 expression was demonstrated between the analyzed races in female tumors—that is, breast invasive carcinoma, ovarian serous cystadenocarcinoma, and uterine corpus endometrial carcinoma—as well as in kidney renal clear cell carcinoma and esophageal carcinoma. Interestingly, ovarian serous cystadenocarcinoma showed statistically significant NACC1 expression in all analyzed races when compared to normal tissue. Due to the difference in NACC1 expression depending on race in the above-described tumors, the use of NACC1 as a potential therapeutic or diagnostic biomarker may be of less importance. Additionally, due to high migration rates worldwide, verifying affiliation to a given race during treatment is virtually impossible. In the analysis of sex, differences in NACC1 expression were presented by the following cancers: kidney renal clear cell carcinoma, kidney renal papillary cell carcinoma, and liver hepatocellular carcinoma. Furthermore, KIRC showed no expression of NACC1 in males, and similarly for KIRP in females. The remaining cancers showed the same statistical significance in males and females. Therefore, KIRC, KIRP, and LIHC, due to their differences in expression with regard to gender, have low utility regarding the use of NACC1 as a diagnostic and therapeutic biomarker. The expression of NACC1 differed in patients by TP-53 mutant status (vs. TP-53 non-mutant) for ESCA, HNSC, LIHC, LUAD, and PAAD tumors. Furthermore, the same tumors expressed NACC1 in patients with and without TP-53 mutants. The expression of NACC1 also differed between normal tissue and tumors, regardless of body weight, in the same cancers (i.e., BLCA, CESC, CHOL, COAD, ESCA, and LIHC). Moreover, in COAD, the expression of NACC1 differed in the comparisons of normal weight (NW) vs. extreme weight (EW), obese (O), and extreme obese (EO) groups. COAD is a tumor associated with non-healthy lifestyles, meaning that COAD has a connection with obesity and high carbohydrate intake. Therefore, this fact confirms the significant role of NACC1 in the development of colon cancer. In the analyzed tumors, no significant differences were demonstrated between the status of lymph node metastases and normal tissue. In several tumors (i.e., ESCA, LUSC, UCEC GBM, COAD, KIRP, CESC, and SARC), NACC1 expression varied depending on age, which, unfortunately, is not positive information for a potential diagnostic or therapeutic factor. A positive aspect of NACC1 expression in relation to the stage of clinical advancement is the lack of differences between the individual stages of clinical advancement in most tumors. Moreover, differences in NACC1 expression occurred in the comparison between normal tissue and tissues of all stages in all the analyzed tumors. The expression of NACC1 was not demonstrated in several tumors in the stage IV tumors (i.e., CHOL, KICH, KIRC, KIRP, PAAD, and READ). Interestingly, KIRP and READ showed expression only in one stage: READ in stage I and KIRP in stage III. This means that NACC1 is important in every tumor stage, which is important for cancer therapy and significant for etiopathogenesis and further research in this direction. In most of the analyzed tumors, no statistically significant differences in NACC1 expression were observed between the tumor grades. The greatest differences were shown for KIRC. This also emphasizes the role of NACC1 in the development of malignant tumors, regardless of the grade of the tumor. We did not find such an extensive and detailed analysis of NACC1 expression in malignant tumors in the available literature and, so, this can be considered an innovative study. Only articles on selected tumors are available in the literature. Therefore, the role of NACC1 in individual tumors is additionally presented below in correlation with the available literature.

Studies on NAC1 in breast cancer have focused mainly on the triple-negative breast cancer (TNBC) subtype, as this is a particularly lethal subtype of breast cancer. High NAC1 expression correlates with poorer prognosis in TNBC. The downregulation of NAC1 reduces markers of cancer stem cells (CSCs) and tumor cell proliferation, migration, and invasion. Furthermore, NAC1 affects immunosuppressive signals, such as transforming growth factor beta (TGFβ) and Interleukin 6 (IL-6), and oncogenic pathways, such as the CD44–JAK1–STAT3 axis. The effect of NAC1 on tumor growth varies depending on the immune status of the host [15]. Ngule et al. demonstrated that five genes (CDK1, EZH2, CCNB1, CCNA2, and AURKA) are involved in cell regeneration. These genes were positively correlated with tumor hypoxia status and the enhanced infiltration of immunosuppressive cells. Furthermore, depletion of the transcriptional cofactor NAC1, which is highly expressed in TNBC, reduced the expression of these genes [16].

In the TCGA database, two correlation analyses were performed for the comparison of the expression levels of NACC1 between breast cancer types. In the first analysis, no differences in the expression of NACC1 were demonstrated between breast cancer types. In the second analysis, TNBC was divided into seven subgroups: TNBC Basal-like 1, (TNBC-BL1), TNBC immunomodulatory (TNBC-IM), TNBC mesenchymal stem-like (TNBC-MSL), TNBC Basal-like 2 (TNBC-BL2), TNBC mesenchymal (TNBC-M), TNBC luminal androgen receptor (TNBC-LAR), and TNBC unspecified (TNBC-UNS). A difference in the expression of NACC1 was demonstrated in the luminal vs. TNBC-UNS, HER2Pos vs. TNBC-UNS, TNBC-BL1 vs. TNBC-BL2, TNBC-BL2 vs. TNBC-MSL, and TNBC-BL2 vs. TNBC-UNS comparisons. In subsequent analyses in the TCGA databases, breast cancer was treated as one group, without division into types. NACC1 expression was demonstrated in breast cancer patients of all races. Moreover, a difference in NACC1 expression was demonstrated between Caucasians and African Americans. Regarding the stage, differences in NACC1 expression were demonstrated for stages 1 vs. 2, N vs. 1, N vs. 2, N vs. 3, and N vs. 4. Tumor grades were not analyzed. The comparison of nodal metastasis status showed a difference in the expression of NACC1 for N vs. N0, N vs. N1, N0 vs. N2, and N0 vs. N3. In the context of breast cancer, there were no associations between gender, age, and TP-53 mutation with the expression of NACC1.

In pancreatic cancer (PAAD), the RNA-binding protein FUS (FUS)-induced circular RNA (circRNA), circRHOBTB3, has been shown to aberrantly inhibit NACC1/Akt/mTOR signaling. This acts as a molecular sponge for miR-600, which then promotes autophagy for PDAC proliferation [11]. In the TCGA database, NACC1 expression has been demonstrated in PAAD. Expression was only demonstrated in Caucasians and Asians when compared to normal tissue. NACC1 expression was only demonstrated in the comparison between normal and stage II tissues. NACC1 expression was demonstrated in PAAD in correlation with the status of lymph node metastasis for N vs. N0 and N vs. N1. Moreover, the expression of NACC1 was demonstrated only in elderly patients (i.e., over 60 and up to 80 years of age) in PAAD. No differences were demonstrated between races, gender, body weight, TP53-mutant, and tumor grades.

NAC1, likely through the regulation of identified signature genes, plays a role in the immunosuppressive nature of the tumor microenvironment (TME). The expression of these genes in hepatocellular carcinoma (HCC) correlates positively with the infiltration of dendritic cells, macrophages, and neutrophils. Additionally, it can predict the responses of HCC patients to anti-PD-1, anti-PD-L1, and anti-CTLA-4 antibodies [17]. In HCC cell lines (i.e., Huh7 and HepG2), the increased expression of NACC-1 was demonstrated, and the downregulation of NACC-1 resulted in decreased cell proliferation and invasion, as well as increased susceptibility to doxorubicin-dependent chemosensitivity. The overexpression of miR-760 in HCC cell lines rescued NACC-1-dependent migration and invasion. Additionally, miR-760 regulates NACC-1 expression in HCC [18]. We observed the expression of NACC1 in primary tumors in comparison with normal samples. The expression levels of NACC1 differed in the HCC patients for stages I vs. II and I vs. III. The expression levels of NACC1 were also associated with nodal metastasis status and differed in the N vs. N0 comparison. No differences were observed for NACC1 with respect to tumor grade, race, age, or weight, while the expression levels of NACC1 differed in the HCC patients by gender and status of the TP-53 mutation.

Interestingly, a rare primary liver tumor has been described based on the fusion of the NACC1 gene with Nipped-B-like protein (NIPBL), previously described as a “cholangioblastic variant of intrahepatic cholangiocarcinoma”. Two such cases have been described in the literature, and a third was found in the TCGA database. The tumors had a characteristic immunoprofile characterized by diffuse inhibin labeling and patchy chromogranin, synaptophysin, and cytokeratin 19 labeling. RNA sequencing in both cases revealed an identical fusion of NIBPL exon 8 with NACC1 exon 2. The NIPBL–NACC1 fusion represents the third type of gene fusion identified in intrahepatic cholangiocarcinoma and correlates with its characteristic morphology [19]. The TCGA database demonstrated NACC1 expression in HCC. There was no difference in NACC1 expression by race, age, body weight, grade, or number of lymph node metastases. There was a difference in expression for the stage I vs. II and stage I vs. III comparisons. Additionally, there were differences in the expression of NACC1 between females and males, as well as by TP-53 mutation status.

NACC1 is regulated by miR-331-3p, which promotes cell proliferation and is involved in urothelial cancer (UC) cell migration and invasion. NACC1 is one of several presumed B1 target molecules of miR-331-3p. Studies have been performed using the urothelial cancer cell lines T24, UMUC6, and KU7. NACC1 expression was assessed in UC derived from a transurethral resection of a bladder tumor (TUR-Bt). In UC cells, cell proliferation was reduced after transient transfection with miR-331-3p precursor and/or NACC1 siRNA. Cell senescence led to cell cycle arrest in the G1 phase through NACC1 inhibition. Additionally, NACC1 inhibition induced the ability of cells to invade and migrate. As a result, 70% of UC cells showed strong positive results for NACC1. Moreover, NACC1 expression was higher in non-invasive UC cells compared to invasive UC cells [20].

In ovarian cancer cells, NAC-1 expression is higher in effusions when compared with their solid tumor counterparts. NAC-1 is upregulated in cancer cells after chemotherapy, suggesting a role of this protein in tumor progression and in the development of resistance to chemotherapy in ovarian cancer [21]. NAC1 silencing was associated with the upregulation of apoptotic genes and downregulation of genes involved in cell movement, proliferation, Notch signaling, and epithelial–mesenchymal transition [22]. Subsequent studies have demonstrated an NAC1-related mechanism of docetaxel resistance in ovarian cancer. NAC1 has a nuclear export signal (NES) at its N terminus (aa 17–28). NES was shown to critically contribute to the shuttling of NAC1 between the nucleus and cytoplasm when ovarian cancer cells were treated with docetaxel. Mechanistically, nuclear-exported NAC1 binds to cullin3 (Cul3) and cyclin B1 via its BTB and BOZ domains, respectively, and the cyto-NAC1-Cul3 E3 ubiquitin ligase complex promotes the ubiquitination and degradation of cyclin B1, hence facilitating mitotic exit and causing cellular resistance to docetaxel [23]. NAC1/FASN overexpression is critical for the growth and survival of a subset of OCCC. FASN silencing by C75-induced phenotypes is dependent on the expression status of the target cell line. Growth inhibition was observed in C75-treated FASN-overexpressing cancer cells compared with the response in FASN-deficient cancer cells [24]. Considering the TCGA database, there has been no comparative analysis of NACC1 expression between normal tissue and ovarian cancer. Furthermore, there have been no analyses of the effects of the number of lymph node metastases, gender, and body weight on NACC1 expression. In this study, a statistical difference in NACC1 expression was observed between all races in ovarian cancer. There was no statistical significance between the tumor stage, age, and TP-53 mutation. There was a statistical difference in the G2 vs. G3 comparison.

NACC1 expression was increased in nasopharyngeal cell lines. Through inhibiting the activation of the AKT/mTOR signaling pathway, NACC1 silencing effectively inhibited nasopharyngeal cell proliferation, migration, and invasion [36]. The TCGA database demonstrated NACC1 expression in HNSCs when compared to normal tissue. The NACC1 expression level in HNSC was independent of race, gender, and age. Differences in NACC1 expression were demonstrated in stage III vs. IV, N vs. I, N vs. II, N vs. III, and N vs. IV comparisons. Additionally, differences in NACC1 expression were demonstrated in the G1 vs. G2, N vs. G1, N vs. G2, N vs. G3, and N vs. G4 comparisons. Differences in NACC1 expression were also demonstrated for the number of lymph node metastases (i.e., N vs. N0, N vs. N1, N0 vs. N2, and N0 vs. N3).

NAC1 expression in melanoma cells is described as essential for immune evasion [29]. Furthermore, NACC1 expression negatively regulates the suppressive activity of memory CD8+ T cells and Tregs [37,38].

NACC1 was overexpressed in four melanoma cell lines compared with primary cultures of normal human epidermal melanocytes, and NACC1 knockdown significantly reduced the migratory activity of all melanoma cell lines. Moreover, it induced an increase in the acetylation status of the cortactin (CTTN) protein. The acetylation status of CTTN, modulated by the NACC1–HDAC6 deacetylation system, induces the acceleration of melanoma cell migration activity via an actin-dependent cellular process. This may be associated with a worse prognosis of melanoma [39]. NAC1 is a negative regulator of NF-κB signaling. As such, NAC1 depletion enhances the level of the nuclear NF-κB in human melanoma. Furthermore, the inhibition of NF-κB signaling increased the antineoplastic activity of NAC1 inhibition in both xenograft tumors and cultured melanoma cells [40]. In the TCGA database, there has been no comparative analysis of NACC1 expression between normal tissue and melanoma, likely due to the lack of a comparison group. There was a difference in NACC1 expression for stage I vs. II. There were no statistical differences in the nodal metastasis status, gender, age, TP-53 mutation, and body weight comparisons.

Limitations of the UALCAN database include the lack of treatment data, patient-reported outcomes, cause of death, local control, disease-free survival, and lack of complications data after treatment. Furthermore, the same clinicopathological parameters are not analyzed in all tumors. In addition, we do not know in detail the level of NACC1 expression depending on the detection method.

## 4. Material and Methods

### 4.1. Databases

To assess the expression levels of NACC1 in 29 different cancers and in normal samples, we used the University of Alabama at Birmingham Cancer (UALCAN) database, which is included in the TCGA project databases. The cancers analyzed included adrenocortical carcinoma (ACC), bladder urothelial carcinoma (BLCA), brain lower-grade glioma (LGG), breast invasive carcinoma (BRCA), cervical squamous cel carcinoma (CESC), cholangiocarcinoma (CHOL), colon adenocarcinoma (COAD), esophageal carcinoma (ESCA), glioblastoma multiforme (GBM), head and neck squamous cell carcinoma (HNSC), kidney chromophobe (KICH), kidney renal clear cell carcinoma (KIRC), kidney renal papillary cell carcinoma (KIRP), liver hepatocellular carcinoma (LIHC), lung adenocarcinoma (LUAD), lung squamous cell carcinoma (LUSC), mesothelioma (MESO), ovarian serous cystadenocarcinoma (OV), pancreatic adenocarcinoma (PAAD, pheochromocytoma and paraganglioma (PCPG), prostate adenocarcinoma (PRAD), rectum adenocarcinoma (READ), sarcoma (SARC), skin cutaneous melanoma (SKCM), stomach adenocarcinoma (STAD), testicular germ cell tumor (TGCT), thymoma (THYM), thyroid carcinoma (THCA), and uterine corpus endometrial carcinoma (UCEC). Moreover, differences in the NACC1 expression levels (RPM, reads per million)—depending on clinicopathological parameters including sample type, race, gender, age, cancer stage, tumor grade, nodal metastasis status, and *TP53* mutation status—of the 29 different cancers were determined and analyzed (Table 2).

The data used and shown in this research are openly available from the TCGA-based databases and do not violate any copyrights [26].

### 4.2. Statistical Analysis

We used website-generated statistical analyses from the UALCAN database and GraphPad Prism 9 (GraphPad, San Diego, CA, USA) for the achieved statistical results and generation of heatmaps. The UALCAN database uses PERL-CGI, a set of Perl modules and scripts that allow you to create web applications and Common Gateway Interface (CGI) scripts, with high-quality graphics using javascript and CSS (Clinical Sample Selection) methods. PERL-CGI is a set of Perl modules and scripts that allow you to create web applications and Common Gateway Interface (CGI) scripts. With Perl-CGI, you can generate dynamic webpages that can interact with users and process form data. Based on it, the expression of the “called gene” is calculated using the desired set of data taken from the TCGA repository (RNAseq data and clinical–pathological patients’ data), and it is generated as the graphical and numerical set of results with statistical analyses. The T-test, Mann–Whitney U-test, ANOVA test, as well as differential expression tests (DEGs) were used. Data normality was estimated using the Shapiro–Wilk normality test. All tests were performed as two-tailed and considered significant at *p* < 0.05, as described previously [41,42].

## 5. Conclusions

While NACC1 is present in many tumors, in some tumors, the expression of NACC1 varies with race, age, body weight, stage, and TP-53 mutant status. Such large fluctuations in NACC1 expression between various cancers further suggest that it is not a good candidate as a diagnostic marker. A diagnostic marker must be reliable, and there is no room for error. However, research on the associated therapeutic effects should be expanded. NACC1, which is expressed in a large number of malignancies, may be one of the key factors involved in the development of tumors. Due to its common occurrence, targeted therapy could be used when its expression is detected in a given tumor. Therefore, further studies are required to better assess the therapeutic potential of NACC1 in more detail.

## Figures and Tables

**Table 1:** The expression levels of nucleus accumbens associated protein 1 (NACC1) in different cancers based on TCGA data. Data taken from the UALCAN database. A *p*-value of <0.05 was considered significant.

Overexpression of NACC1 in Cancers
bladder urothelial carcinoma breast invasive carcinoma cervical squamous cell carcinoma cholangiocarcinoma colon adenocarcinoma esophageal carcinoma glioblastoma multiforme head and neck squamous cell carcinoma kidney chromophobe kidney renal clear cell carcinoma liver hepatocellular carcinoma lung adenocarcinoma lung squamous cell carcinoma pancreatic adenocarcinoma prostate adenocarcinoma stomach adenocarcinoma thyroid carcinoma uterine corpus endometrial carcinoma

**Table 2:** Patient characteristics.

No.	TCGA Dataset	Number of Samples	Female	Men
1.	Adenocritical carcinoma	79	48	31
2.	Bladder urothelial carcinoma	402	297	105
3.	Brain lower-grade glioma	514	229	285
4.	Breast invasive carcinoma	1087	1075	12
5.	Cervical squamous cell carcinoma	305	305	0
6.	Cholangiocarcinoma	36	20	16
7.	Colon adenocarcinoma	283	127	156
8.	Esophageal carcinoma	183	26	157
9.	Glioblastoma multiforme	155	54	101
10.	Head and neck squamous cell carcinoma	519	136	383
11.	Kidney chromophobe	65	27	38
12.	Kidney renal clear cell carcinoma	533	188	345
13.	Kidney renal papillary cell carcinoma	290	76	214
14.	Liver hepatocellular carcinoma	362	117	245
15.	Lung adenocarcinoma	514	276	238
16.	Lung squamous cell carcinoma	494	128	366
17.	Mesothelioma	87	16	71
18.	Ovarian serous cystadenocarcinoma	302	302	0
19.	Pancreatic adenocarcinoma	177	80	97
20.	Pheochromocytoma and paraganglioma	179	101	78
21.	Prostate adenocarcinoma	497	0	497
22.	Rectum adenocarcinoma	165	75	90
23.	Sarcoma	259	118	141
24.	Skin cutaneous melanoma	461	175	286
25.	Stomach adenocarcinoma	415	147	268
26.	Testicular germ cell tumors	128	0	128
27.	Thymoma	120	57	63
28.	Thyroid carcinoma	505	369	136
29.	Uterine corpus endometrial carcinoma	546	546	0

## Data Availability

The datasets used during the current study are available online from the UALCAN database, or from the corresponding author on reasonable request.
